# Linear and nonlinear record high optical birefringence in anisotropic van der Waals crystals

**DOI:** 10.1038/s41377-024-01662-4

**Published:** 2025-01-01

**Authors:** Luca Sortino

**Affiliations:** Chair in Hybrid Nanosystems, Faculty of Physics, Ludwig-Maximilians-Universität München, 80539 Munich, Germany

**Keywords:** Nonlinear optics, Optical materials and structures

## Abstract

Multilayered van der Waals (vdW) materials are semiconductors composed of atomically thin crystal layers, held together by weak vdW forces. They offer unique crystal structures and electronic properties, distinct from conventional semiconductors, making them a promising platform for linear and nonlinear optics. In this context, the large refractive indexes given by highly polarizable transition metals, combined with excitonic resonances and unconventional crystalline structures, provides a toolbox for exploring non-linear physics and strong light–matter interactions with unprecedented opportunities for nanoscale optics. Recent reports highlight novel vdW materials, particularly PdPSe, a pentagonal crystal with strong nonlinear responses, and As_2_S_3_, a record high birefringence crystal, as favorable candidates to engineer nonlinear responses and miniaturization of optical components, owing to the combination of high refractive index and strong optical anisotropy of the underlying crystal structures. While still in its infancy, research on vdW materials promise a florid ground for fundamental studies, bridging the gap between material science and nanoscale optics.

Nanomaterials are a fundamental platform to advance optical devices and develop novel technologies. In recent years, van der Waals (vdW) crystals, particularly as single-layered, two-dimensional (2D) materials, have captivated the scientific community with their extraordinary characteristics. Their layered nature of atomically thin layers presents unique electronic, optical, and mechanical properties. In particular, the strong excitonic and nonlinear responses make them ideal candidates for next-generation devices from light emission to frequency converters, optical switches, and ultrafast lasers. Today, significant efforts are being made in foundries worldwide to integrate 2D materials into industrial production lines. Likewise, fundamental research has established these materials across a range of fields, from nano electronics to nano optics, to catalysis and quantum materials. Beyond their 2D form, multilayers of vdW materials, such as transition metal dichalcogenides (TMDCs), emerged as a promising class of dielectric platform for linear and nonlinear photonics. Combined with strong excitonic resonances up to room temperature, vdW multilayers opens to unprecedented design opportunities.

Aided by the layered structure and transition metals with high polarizability, vdW materials exhibit high refractive indexes^1^, which allows to generate intrinsic optical resonances, from Fabry–Perot types, to geometrical resonances in nanostructured thin films such as waveguides, optical resonators and metasurfaces^2^. Together with a vast library of different compounds to explore, vdW materials can outperform conventional platforms and amplify the potential design of optical metamaterials. Moreover, a significant advantage is their ability to be manipulated into vertical heterostructures with atomic precision owing to a general lack of lattice mismatch restrictions. Stacked vdW layers are a route for highly customized light–matter interaction and control over light phase propagation and nonlinear efficiencies. The lack of crystalline selectivity gives the ability to twist each vdW layer, opening a vast landscape of design possibilities where each layer’s material, orientation, and thickness can be tailored to achieve specific optical responses^3^.

In this context, strong optical anisotropies are essential to manipulate light and to control phase and polarization properties. If a material exhibits a strong one-dimensional component in its atomic geometry, for instance looking at a vertically layered material from its side, the propagation of the polarization component of light oriented either parallel or perpendicular to the crystal axes experiences a different electromagnetic response, and as such a difference in their phase and propagation length is accumulated along the thickness of the material, leading to the common effect of birefringence (Fig. 1a). This difference in phase at the output can be harnessed for light modulation and nonlinear optics, and the discovery of novel materials for miniaturization could find use in ultracompact optical components and interferometers. Two recent works published in *Light: Science & Applications* highlight novel vdW crystals with unprecedented optical anisotropies for both linear and nonlinear optical processes^4,5^.

**Fig. 1:**
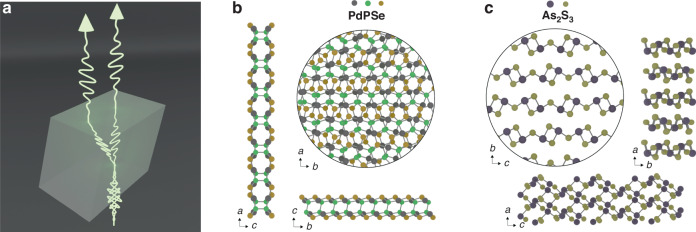
Optical birefringence in novel van der Waals crystals. **a** Optical birefringence occurs when light travels through an anisotropic material, such as a crystal with a unique internal structure that causes different light beams (polarized in different directions) to travel at different speeds. This results in the splitting of incident unpolarized light beam into two polarized output rays: the ordinary and the extraordinary rays, each experiencing different indices of refraction. **b** Axis projection of the crystalline structure of the pentagonal crystal structure of single layer PPdSe. **c** Axis projection of the crystal structure of As_2_S_3_

The work^4^ by Zhu et al. investigated PdPSe, a pentagonal vdW crystal for anisotropic nonlinear light generation (Fig. 1b). The results combine a high refractive index material with large second and third order susceptibilities, leading to intense polarization properties via the intrinsic anisotropy. PdPSe belongs to the family of TMDCs, however its unique crystal structure is composed of two sub-layers forming a single monolayer with pentagonal structure. Here, the breaking of inversion symmetry for second-order processes is observed only in even-layered crystals, opposite to common TMDCs, such as MoS_2,_ for which even-layers restore the inversion symmetry. The strong correlation between crystal structure and nonlinear light generation is shown to provide an extremely strong second harmonic generation along its main axis. Moreover, the dispersion of the nonlinear susceptibility provides a strong wavelength-dependent anisotropy, opening to potential applications in nonlinear imaging.

A broader analysis of the large anisotropy values in vdW materials is discussed in the second work by Slavich et al.^5^. Here the authors found As_2_S_3_, a monoclinic layered crystal (Fig. 1c), as the best candidate to achieve unprecedented values of in-plane optical birefringence, overcoming by a factor of 20% the values of century-long record set by rutile. The study sets to find the largest geometrical anisotropy starting from symmetry observation in the elementary crystal cell of layered materials. Amongst the ones combining high refractive indexes and large anisotropy, As_2_S_3_ stands out as the candidate for achieving large birefringence in visible range, owing to a bandgap at 460 nm (2.7 eV). Optical characterization of the material demonstrates large birefringence values, Δ*n* ~ 0.4, outperforming common crystals. To further demonstrate the advantages of employing high refractive index materials, the authors show the operation of a thin As_2_S_3_ crystal as a quarter waveplate where multiple Fabry–Perot reflections confer an additional retardance to the light propagation, unlocking the possibility to further reduce the material thickness—down to only 345 nm—for a true operational zero order waveplate at visible wavelengths.

In summary, exploiting the inherent anisotropies and high refractive indexes in vdW materials opens to the development of advanced photonic devices. The advantages lie in an unprecedented precision on the intrinsic atomic structure of devices realizing vdW heterostructures, and the control on light–matter interactions at the nanoscale, by engineering optical resonances via intrinsic resonances in the material. As the field progresses, several challenges remain, from understanding the fundamental mechanisms, optimizing fabrication techniques for large-scale production, and integrating vdW materials with existing technologies. Nevertheless, the rapid pace of discovery in this area suggests that vdW materials are a promising platform to make a significant impact on the future of linear and nonlinear optics.
